# Calcific Inflammation

**Published:** 1889-04

**Authors:** J. G. Templeton

**Affiliations:** Pittsburg, Penn.


### Calcific Inflammation.

BY J. G. TEMPLETON, D.D.S., PITTSBURG, PENN.

Read before the Mississippi Valley Dental Society, Cincinnati, March 7th, 1889.

When I first saw the programme for this meeting and that my name was on it for a paper, my first thought was that after over twenty-two years experience in combatting that kind of trouble, I can tell them all I know about it in less than two minutes.

My next thought was that it might perhaps, be better for me to conform to the "innocuous desuetude" and treat the subject a little more elaborately.

Calcific inflammation, is a term used to express the origin of an inflamed and hemorrhagic condition of the margins of the gums so frequently seen in our practice. When such an abnormal state is found, the cause is usually so obvious, that any word alluding to its origin might be left out of the nomenclature. We therefore prefer as a name for this disease, the words hemorrhagic ulitis, but as I do not consider myself authority on nomenclature, "I add not," as some of the old ministers used to say at the end of their long sermons.

"Pyorrhœa alveolaris" is an appropriate name where the conditions warrant its use, but such cases are few in comparison with the great number properly classed as calcific inflammation and phagadenic pericementitis, (according to Dr. Black). And here we are inclined to think that perhaps the majority of our profession fail to differentiate, and also often use these terms interchangeably as if "tweedledee and tweedledum" were without variation.

For a concise and lucid description with erudite discrimination, and also for the beautiful and remarkably correct illustrations of this lesion of the oral cavity, we take pleasure in referring you to the chapter by Prof. Black on this subject in vol. 1 of the "*American System of Dentistry*." We shall therefore not weary your patience with any attempt to describe any peculiar phase of the disease under consideration. We wish, however, to call your attention particularly to what may be considered as a primary factor in the etiology of a large percentage of these abnormal conditions.

We refer now to its scorbutic origin and of its having some symptoms in common with scurvy, though differing in intensity and duration, and if we compare these departures from the normal state, either systematically or locally, we do not find petechiæ or vibices in connection with the malady which now engages our attention, but we often see what may be called a mild form of, or partial ecchymosis, the visible signs of which are observed in the pale yellow, or saddle-flap color of the cuticle; there is in both the same tendency towards loosening and loss of the teeth and also hemorrhage from the gums, more or less profuse from slight causes.

Scorbutic affections are generally associated in our minds with long sea voyages and their treatment has, by the people, been considered as coming within the prerogative of a physician; hence the paucity of our professional literature in reference thereto, while that of the medical profession is quite extensive. So much so that Irving C. Rosse says in the reference hand-book of the medical sciences that, "to write the history of scurvy would almost be to write the history of medicine and to chronicle the sanitary circumstances of most human events, since the disease has occurred from time immemorial, both on sea and on land. That it exists on land proofs are abundant.

Hammond says (in report on scurvy U. S. Sanitary Commission) "that at Council Bluffs in 1820, nearly the entire garrison was attacked and many died, and the efficiency of the U. S. forces in the Florida and Mexican wars was very materially lessened by its occurrence." It existed to a very great extent in the English and French armies in the Crimea, and it also added largely to the mortality during our late war. In reference to these statements Prof. Flint says, "The inconsistency between these facts and the existing state of knowledge, is in part to be explained by the inability always to secure the means of prevention in military operations, but it is in a greater measure to be accounted for by a censurable ignorance or neglect of these means." The treatment for scurvy, whether preventative or curative, is almost entirely dietetic and consists of fresh meats and all kinds of vegetables.

That calcific inflammation, phagedenic pericementitis and pyorrhœa alveolaris simulate scurvy, the writer has no doubt, and in his practice has for many years insisted on the use of the same dietetic regimen, and once in five days the application of an agent that is caustic, astringent, and stimulating, with occasional use of mild laxatives to correct constipation. No other systemic medicine being necessary. And we will say here that when these instructions have been faithfully observed by the patient we have seen the pale yellow, tawny, or septicæmic appearance of the face changed to almost the flush of youth in from four to eight weeks. But to maintain this condition, the care necessary to be observed on the part of the patient in all *bad cases* is like the " price of liberty."

The writer's treatment consists of the removal of all calcareous or serumal deposits, arresting all hemorrhage with hot water and then apply pure and finely pulverized sulphate of copper, which is also washed out with hot water, repeating the same locally as previously stated, until no longer necessary, and we find that the fifth application is rarely required.

From the commencement of the treatment we impress upon the minds of our patients the importance of cleansing the mouth with soap, as the most necessary part of their toilet preparations, before partaking of the morning meal. Also giving specific directions in reference to an anti-scorbutic diet, viz: fresh meats, a regular use of pickles of any kind, a free use of lemons and oranges, and also the daily use of fresh vegetables, consisting of one or more of the following, viz: onions, cabbage, turnips, radishes, horse-radish, mustard, water-cresses, lettuce, spinach, celery, parsley, parsnips, carrots, potatoes, and sauer-kraut.

We know as articles of diet onions and sauer-kraut are very much tabooed by many, especially the elite, but in our humble opinion, their affluvia is superlatively delectable in comparison with the odor coming from the mouths of many persons having this disease.

